# Forecasting standardized precipitation index using data intelligence models: regional investigation of Bangladesh

**DOI:** 10.1038/s41598-021-82977-9

**Published:** 2021-02-09

**Authors:** Zaher Mundher Yaseen, Mumtaz Ali, Ahmad Sharafati, Nadhir Al-Ansari, Shamsuddin Shahid

**Affiliations:** 1 Institute of Research and Development, Duy Tan University, Da Nang, 550000 Vietnam; 2 Deakin-SWU Joint Research Centre on Big Data, School of Information Technology, Deakin University, Burwood, VIC 3125 Australia; 3 Department of Civil Engineering, Science and Research Branch, Islamic Azad University, Tehran, Iran; 4 Civil, Environmental and Natural Resources Engineering, Lulea University of Technology, 97187 Luleå, Sweden; 5 Department of Water and Environmental Engineering, School of Civil Engineering, Faculty of Engineering, Universiti Teknologi Malaysia (UTM), 81310 Skudai, Johor Malaysia

**Keywords:** Climate sciences, Climate change, Projection and prediction, Hydrology

## Abstract

A noticeable increase in drought frequency and severity has been observed across the globe due to climate change, which attracted scientists in development of drought prediction models for mitigation of impacts. Droughts are usually monitored using drought indices (DIs), most of which are probabilistic and therefore, highly stochastic and non-linear. The current research investigated the capability of different versions of relatively well-explored machine learning (ML) models including random forest (RF), minimum probability machine regression (MPMR), M5 Tree (M5tree), extreme learning machine (ELM) and online sequential-ELM (OSELM) in predicting the most widely used DI known as standardized precipitation index (SPI) at multiple month horizons (i.e., 1, 3, 6 and 12). Models were developed using monthly rainfall data for the period of 1949–2013 at four meteorological stations namely, Barisal, Bogra, Faridpur and Mymensingh, each representing a geographical region of Bangladesh which frequently experiences droughts. The model inputs were decided based on correlation statistics and the prediction capability was evaluated using several statistical metrics including mean square error (*MSE*), root mean square error (*RMSE*), mean absolute error (*MAE*), correlation coefficient (*R*), Willmott’s Index of agreement (*WI*), Nash Sutcliffe efficiency (*NSE*), and Legates and McCabe Index (*LM*). The results revealed that the proposed models are reliable and robust in predicting droughts in the region. Comparison of the models revealed ELM as the best model in forecasting droughts with minimal *RMSE* in the range of 0.07–0.85, 0.08–0.76, 0.062–0.80 and 0.042–0.605 for Barisal, Bogra, Faridpur and Mymensingh, respectively for all the SPI scales except one-month SPI for which the RF showed the best performance with minimal *RMSE* of 0.57, 0.45, 0.59 and 0.42, respectively.

## Introduction

Drought is a natural disaster that affects society and the environment frequently^1,2^. It significantly influences water resources availability, agricultural production, environmental health and thus, socio-economy of a region^3,4^. There is no definite way of defining drought because it is not possible to determine the exact duration of a drought event. Drought slowly builds over time and leaves a prolonged influence over a large geographical space without any significant infrastructural damage^5,6^. The complexity of a drought event is characterized by its duration, intensity, and severity. In a simple term, drought is defined as the period of a temporary shortage of water resources due to persistently low precipitation.

Drought can have different forms, such as meteorological drought, hydrological drought, agricultural drought, and socioeconomic drought^7–9^. Meteorological droughts occur due to deficiency of precipitation from the average. It is the initiator of all other kinds of droughts and therefore, most widely studies for monitoring droughts^10^. Meteorological drought frequency does not depend on the average precipitation of an area, rather the variability of precipitation. The large variability of precipitation on the deficit side indicates droughts. Therefore, it can occur in any climatic regions including tropical region like Bangladesh^11,12^ or humid climate zone like Malaysia^13,14^. Even it can happen in northeast India, the highest rainfall region of the world^15^. A recent study suggests that the wetter parts of the earth would experience more devastating droughts in future^16^. It urges more attention to be given for monitoring and forecasting droughts in tropical regions. Droughts are often more devastating when it occurs in tropical regions as the ecosystem of such region is habituated with high year-around rainfall^17^.

Bangladesh, located in tropical South Asia experienced several devastating droughts due to shortfalls in precipitation^18^. The droughts caused prolonged water shortages and consequently affected agriculture, environment, and health^19^. Factors that intensify the impact of drought include population growth, agricultural expansions, land use changes, and industrial development due to associated increase in water demand^20^. There is a need to have a proper understanding and modeling of drought to ensure sustainable planning and management of water resources. However, the slowly emerging characteristics of droughts causes a challenge in determining and modeling of drought duration, intensity, severity, spatial extent and inter-arrival period^21,22^.

Drought is a common natural disaster in Bangladesh which generally occurs twice in a decade^23^. Agricultural damages from drought are more frequent in the country compared to any other natural disasters^24^. Therefore, a large number of studies have been conducted to characterize meteorological droughts in Bangladesh in recent years^25–28^. Besides studies have been conducted to assess drought risk^18^ and impacts on droughts in agriculture^24^, economy, water resources^29^ and society^30^. However, no studies have been conducted so far to forecast droughts in Bangladesh, though it is highly important for the country from a socio-economic point of view, particularly in the context of climate change.

The rainfall of Bangladesh is changing due to the changes in global climate^18,31^. This has caused a rise in weather extremes^32^ and hydrological disasters^23^ in the country. Mohsenipour et al.^23^ reported an increase in the return period of droughts in highly drought-prone regions of Bangladesh due to rises in temperature and changes in rainfall pattern. More economic damages due to frequent droughts can be anticipated in future due to climate change. The persistent negative impact of drought on water resources and associated water scarcity and economic damages demands the development of models for the effective prediction and monitoring of drought to ensure a proper establishment of strategies for the management of drought-related risks^33–35^. Improper drought prediction always results in poor drought management, hence, there is a need to build fast, reliable and accurate models for drought prediction which can provide quantitative data on impending drought-related risks. With such models, drought episodes can be accurately predicted by anticipating future changes in drought indices based on information derived from current and historical hydro-meteorological data^36–38^.

A large number of DIs has been developed for monitoring droughts^10,26^. Among all, the standardized precipitation index (SPI) is the most simple, statistically robust, comprehensible, and independent of climatic factors^19^. Despite the recent introduction of SPI^39^, it has been widely accepted in the drought prediction community as a useful DI and has been used in numerous studies to investigate drought variability when assessing the impact of drought in agricultural and hydrological sectors^11,26,40^.

Several forecasting models have been introduced for forecasting droughts such as autoregression integrated moving average (ARIMA), multiple linear regression (MLR) and Markov Chain^41^. SPI is a probabilistic index derived from a tailed distribution of rainfall deficit. Therefore, the scale of SPI is not linear. This has made the forecasting of droughts using conventional statistical methods more challenging. Most recently, the applications of machine learning (ML) models have exhibited outstanding progress on modeling drought indices and climatology^42,43^. Several versions of ML models have been developed for SPI forecasting including artificial neural network (ANN), support vector regression (SVR), extreme learning machine (ELM), adaptive neuro-fuzzy inference system (ANFIS), M5 Tree (M5T), random forest (RF), linear genetic programming (LGP), least-square support vector regression (LSSVR), extremely randomized tree (ERT), multivariate adaptive regression spline (MARS), wavelet preprocessing integrated ML models and bio-inspired hybrid ML models^38,44–51^. Although there have been diverse models introduced for modeling DIs, it is difficult for scientists and scholars to determine a generalized or a perfect model that can suit all types of climates. Besides, there is a chance of misleading in model development if the non-appropriate variables of models’ structure are set-up. Furthermore, every region behaves differently following the weather stochastics and historical characteristics.

The current research is devoted to the development of machine learning models for SPI forecasting for Bangladesh. Five different versions of machine learning models were developed (RF, MPMR, M5tree, ELM and OSELM) for forecasting SPI at multiple time-scales (1, 3, 6 and 12 months). One-month SPI indicates a short-period deficit of rainfall which affect ecology, air temperature and public health of the country^52^. Three- and six-month SPIs are used to assess agricultural drought in Bangladesh^53^, while nine- and twelve-month SPIs are responsible for declination of river flow and groundwater level or hydrological droughts. Therefore, models were developed for forecasting SPI of those five time-scales. The models were developed only for four stations (i.e., Barisal, Bogra, Faridpur and Mymensingh), each representing individual climate zone where droughts usually occur in Bangladesh. Among the six major geographical regions of Bangladesh^54^, droughts mostly occur in the north, central, central-north and southwest regions. Therefore, models were developed for forecasting droughts at Barisal, Bogra, Faridpur and Mymensingh representing southeast, north, central and central-north regions of Bangladesh. Historical data of 64 years (1949–2013) was used to develop and validate the model.

## Materials and methods

### Case study

**Figure 1:**
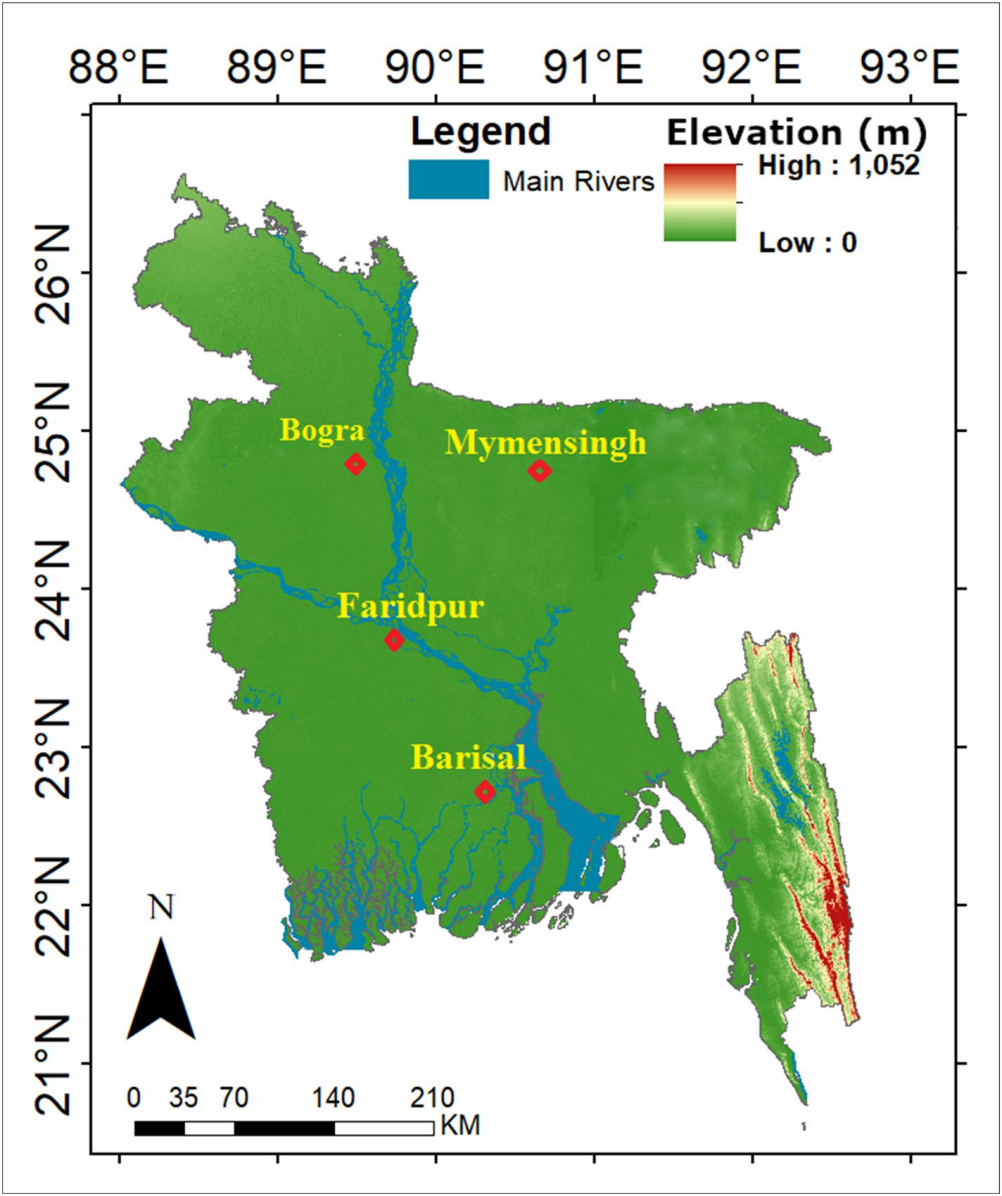
The locations of the studied meteorological stations in Bangladesh.

Bangladesh is located in the deltas of large rivers flowing from the Himalayas covers an area of 144,000 km^2^ (Fig. 1, https://www.diva-gis.org/gdata). A tropical humid climate dominates in most of the country. The minimum temperature of the country goes below 12.8 °C in January while the maximum temperature goes above 31.1 °C in May. Due to an extremely flat topography of the country, the spatial variability of temperature is very low. The orientations of temperature gradient are different for different seasons. Therefore, overall there is very less variability in annual mean temperature among different geographical regions. The rainfall in Bangladesh ranges between 1600 and 4400 mm in the northwest and northeast respectively. Seasonal and annual variability of rainfall is very high. About 75% to annual rainfall occurs in monsoon months of May to September and only 3% rainfall occurs during December–February. The coefficient of annual variability of monsoon rainfall in more than 30% in a major portion of the country. The high variability of rainfall often causes droughts in the country. The country experienced major droughts in the years, 1963, 1966, 1968, 1973, 1977, 1979, 1982, 1989, 1992 and 1994–1995.

### Theoretical overview and SPI calculation

The review of ML models used in this study, RF, MPMR, M5tree, ELM and OSELM are provided in this section.

#### Random Forest (RF)

The RF is created based on the concept of ensemble and bagging learning approach^55^. It uses the decision tree methodology which performs the bagging procedure for solving the regression problem^55,56^. Each node in RF is separated randomly by selecting the most important input predictors to enhance the learning process that leads to better prediction accuracy as well as maintaining the robustness to avoid overfitting^57^. The steps are followed to construct an RF model:

i. Select random k data points from training data.
ii. Construct the decision tree associated with the data in (i).
iii. Choose the n-decision tree (*n*_trees_) that needs to build.
iv. Repeat i and ii.
v. Cumulate the aggregative predictions of *n*_trees_ to forecast multi-scaler SPI.

The capacity of the RF has been approved in modeling different phenomena in atmospheric, hydrological and geosciences engineering^58^, environmental management^59^, drought forecasting^60^, rainfall forecasting^61^, solar index estimation^62^ and most recently forecasting soil moisture^63^.

**Figure 2:**
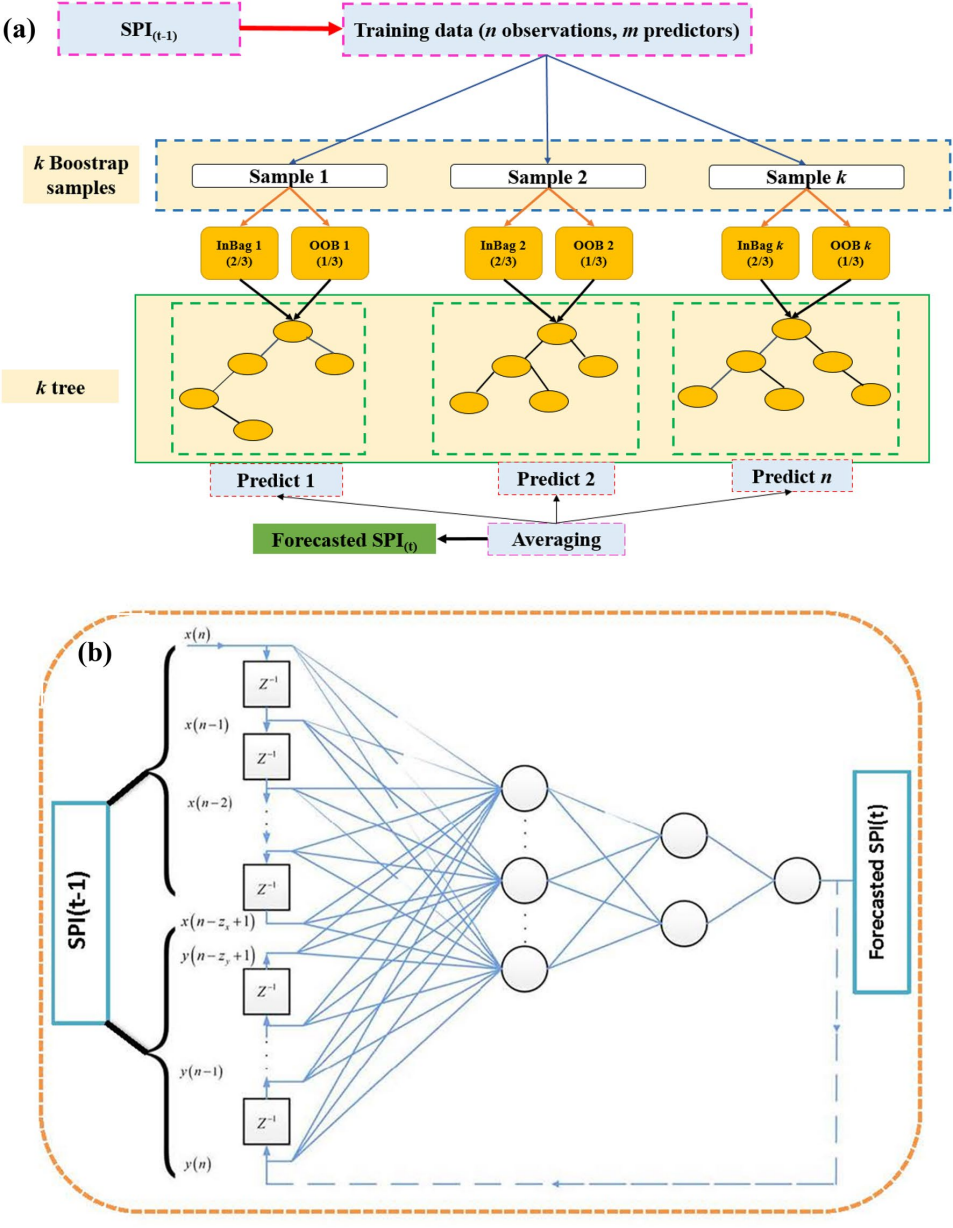

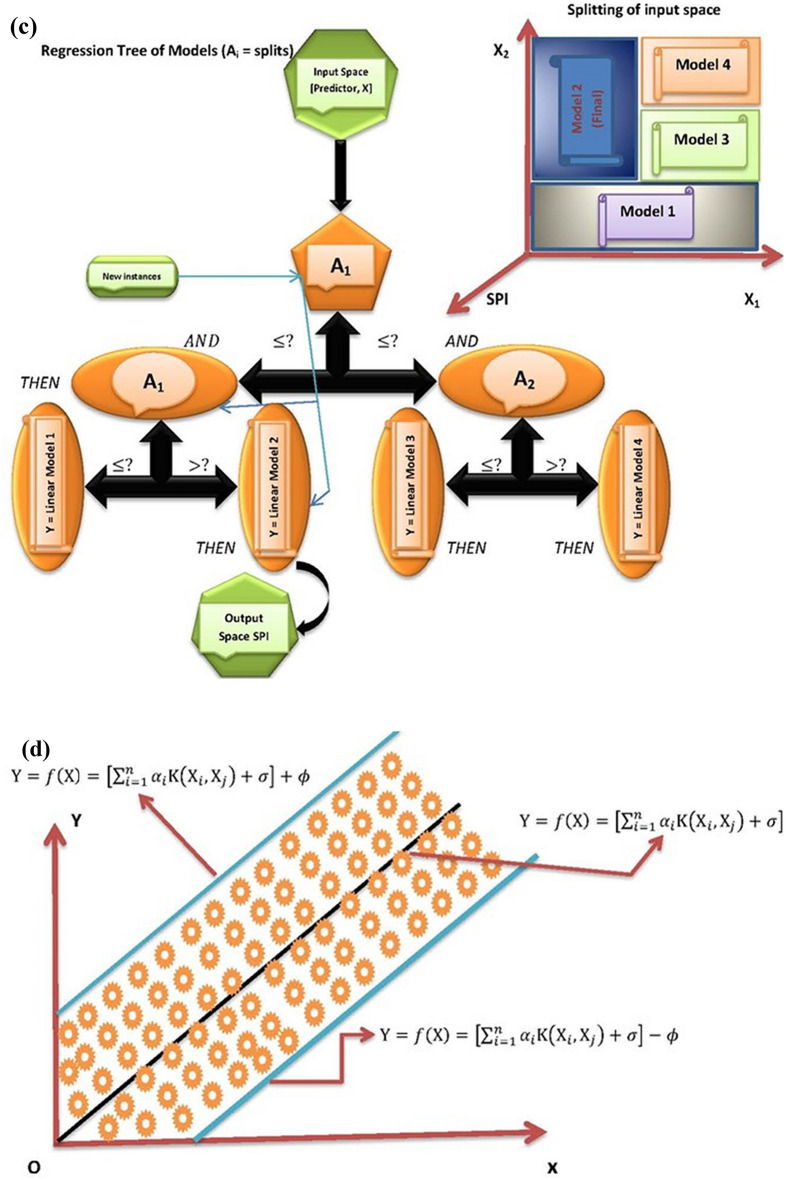
(**a**) Schematic view of RF model, (**b**) Basic structure of ELM model, (**c**) Representation of M5tree model, (**d**) The schematic view of MPMR model.

For more comprehensive studies on RF model, readers are referred to^57,64–66^. The flowchart of the random forest model is provided in Fig. 2a.

#### The extreme learning machine (ELM)

ELM designed by Huang et al. is an advanced data intelligent model that uses Single Layer Feed forward Neural Network (SLFN)^67^. ELM is very fast and more efficient than the existing data-driven models^68^. Mathematically the ELM can be formulated as^69^:

1 $${f}_{M}\left(x\right)={\sum }_{i=1}^{M}{\rho }_{i}{h}_{i}\left({x}_{i}\right)=h\left(x\right)\rho$$

where ${\rho }_{i}= {\left[{\rho }_{1}, {\rho }_{2},\ldots ,{\rho }_{M}\right] }^{T}$ is the output weight vector between the hidden layer of *M* nodes to the m ≥ 1 output nodes, and ${h}_{i}\left({x}_{i}\right)= {\left[{h}_{1}\left(x\right), {h}_{2}\left(x\right),\ldots ,{h}_{M}\left(x\right)\right] }^{T}$ is ELM nonlinear feature mapping and ${f}_{M}\left(x\right)$ is the final ouptput/prediction. For example, the output (row) vector of the hidden layer with respect to the input $x.$ ${h}_{i}\left(x\right)$ is the output of the *ith* hidden node output. The output functions of hidden nodes may not be unique. Different output functions may be used in different hidden neurons. In real life problems ${h}_{i}\left({x}_{i}\right)$ can be written as:

2 $${h}_{i}\left({x}_{i}\right)=G\left({a}_{i},{b}_{i},x\right), {a}_{i}\in {R}^{d}, {b}_{i}\in R$$

where $G\left(a,b,x\right)$ (with hidden node parameters (a, b)) is a nonlinear piecewise continuous function satisfying ELM universal approximation capability theorems and $R$ is the set of real numbers whereas ${R}^{d}$ is the d-dimensional set of real numbers and ${x}_{i}$ is the input data. The commonly used mapping function/activation function in ELM are Sigmoid function, Hyperbolic tangent function, Gaussian function, Hard limit function, Cosine function and Fourier basis functions. ELM trains an SLFN in random feature mapping and linear parameters solving phases. First ELM randomly adjusts the hidden layer to map the input predictor into a feature space with the help of some nonlinear functions. The random feature phase differentiates ELM from SVM and deep neural networks. The nonlinear activation functions are basically nonlinear piecewise continuous functions. The hidden node parameters (a, b) in ELM are randomly created which are independent of the training data.

In the second phase of ELM learning, the weights connecting the hidden layer and the output layer, represented by $\rho$, are solved by reducing the approximation error in the squared error sense:

3 $$\begin{array}{c}min\\ \rho \end{array}\Vert H\rho -T\Vert$$

where $H$ is the hidden layer output matrix which can be simplified as follows^69^.

4 $${\rm H}=\left[\begin{array}{c}h\left({x}_{1}\right)\\ \vdots \\ h\left({x}_{N}\right)\end{array}\right]=\left[\begin{array}{ccc}{h}_{1}\left({x}_{1}\right)& \cdots & {h}_{M}\left({x}_{1}\right)\\ \vdots & \cdots & \vdots \\ {h}_{1}\left({x}_{N}\right)& \cdots & {h}_{M}\left({x}_{N}\right)\end{array}\right]$$

and $T$ is the training data matrix, which can be written as:

5 $$T=\left[\begin{array}{c}{{t}_{1}}^{T}\\ \vdots \\ {{t}_{N}}^{T}\end{array}\right]=\left[\begin{array}{ccc}{t}_{11}& \cdots & {t}_{1m}\\ \vdots & \cdots & \vdots \\ {t}_{N1}& \cdots & {t}_{Nm}\end{array}\right]$$

The ∥ · ∥ denotes the Frobenius norm. The optimum solution to (3) is given by:

6 $${\rho }^{*}={\rm H}^{+}T$$

Here ${\rm H}^{+}$ is the Moore–Penrose generalized inverse of matrix of *H*. The principle which differentiates ELM from the conventional neural network model is that every parameter of the feed-forward networks (input weights and hidden layer biases) is not necessary to be fine-tuned. The SLFNs with randomly selected input weights effectively learn different training patterns with minimum error. Following randomly selecting input weights and the hidden layer biases, SLFNs can be deemed as a linear system. The output weights which connect the hidden layer to the output layer of this linear system can now be systematically solved by generalized inverse operation of the hidden layer output matrices. This makes ELM model many times faster than that of conventional feedforward learning algorithms. The flowchart of ELM model is shown in Fig. 2b.

#### The online sequential extreme learning machine (OSELM)

A standalone ELM which uses all *N*-samples data for the training. However, the data chunk-by-chunk may be used in solving real-world complexity because the process of learning is a very time consuming and requires new data for training ELM each time the model is run^70^. The OSELM performs in two learning stages as the variant of a standalone ELM model, *i.e*., a sequential learning stage and initialization stage. In the initialization stage, for a given training dataset ${\aleph }_{k-1}$:

7 $${\aleph }_{k-1}=\left\{\left({x}_{j},{t}_{j}\right):j=\mathrm{1,2},\cdots k-1\right\}$$

where ${x}_{j}$ is the input data point and ${t}_{j}$ is the jth parameter. The initial output weight is given by:

8 $${\rho }_{k-1}={\theta }_{k-1}{H}_{k-1}^{t}{T}_{k-1}$$

where ${\rho }_{k-1}$ is the initial output weight, ${\theta }_{k-1}={\left({H}_{k-1}^{t}{H}_{k-1}\right)}^{-1}$ is the Moore–Penrose generalized inverse of matrix, ${{H}_{k-1}=\left[{h}_{1}^{t}, \ldots ,{h}_{k-1}^{t}\right]}^{t}$ is the hidden layer output matrix, and ${{T}_{k-1}=\left[{t}_{1}, \ldots ,{t}_{k-1}\right]}^{t}$ is the training data matrix.

The biases and random weights are assigned to the small chunk in the initialization stage to compute the hidden layer output matrix in the initial SPI (W) training data. The sequential learning phase is then initiated where RLS algorithm is employed to update the output weights in a recursive way^70^. The output weights in OSELM are recursively updated based on the intermediate results in the last iteration and the newly arrived data, which is discarded instantly once they have been learnt, and therefore, the calculation overhead and the memory requirement of the algorithm are significantly decreased. The readers can consult literature for further details on OS-ELM^71–73^.

#### The M5 tree (M5tree) model

The M5tree model works on the binary decision tree structure, is an ordered and hierarchical model^74^. The connection is initiated between inputs and output at the terminal nodes using linear regression^75^. Tree-based models are made according to a divide-and-conquer method for establishing a relationship between the inputs and output^76^. Two steps are involved to build the M5tree model i.e. In the first step, the data is partitioned into subsets to create model tree which is based on standard deviation that reaches a node as a measure of error and determining the expected decrease in the error as a result of testing each attribute^77^. The method is recursive in which the data points are divided into subsets similar to a test that depends on the standard deviation and the error depletion ${\lambda }_{R}$ as given^77,78^:

9 $${\lambda }_{R}=\lambda (\Gamma )-\sum \left(\frac{{\Gamma }_{j}}{\Gamma }\lambda ({\Gamma }_{j})\right)$$

where $\lambda$ is set of examples that reach the nodes and ${\Gamma }_{j}$ is a subset of examples that have the $jth$ output of the potential set outputs while ${\lambda }_{R}$ is the standard deviation. Due to branching procedure, data in child nodes have fewer ${\lambda }_{R}$ than parent nodes. A structure is selected that has the maximum expected error reduction by analyzing all possible structures. This dividing and conquering rule frequently produces a great tree-like structure that leads to overfit and to prevent overfitting, the overgrown tree is pruned, and pruned subtrees are substituted with linear regression functions in the second step. General form of the model is^77^:

10 $${\lambda }_{R}= {a}_{0}+{a}_{1}{x}_{1}+{a}_{2}{x}_{2}$$

where ${a}_{0}{, a}_{1}, {a}_{2}$ are the linear regression constants. Figure 2c represents the basic structure of M5tree model.

#### The minimax probability machine regression (MPMR) model

The MPMR is a probabilistic, nonlinear regressor model which increase the least probability in the correct regression interval of the objective function. The MPMR is using convex optimizations and linear discriminant^79^, which make MPMR a good and improved version of Support Vector Machine^79^. The data is calculated among $+\delta$ and $-\delta$ with the axis of a dependent variable by shifting all of the regression data. The boundary between the two is a regression surface, where the upper and lower bounds of probability are identified for misclassifying a point without making distributional assumption^80^. The learning (D-dimensional) inputs are generated from undefined regression as follows:

11 $$f:{\text{ R}}^{{\text{D}}} \to {\text{R}},$$

$$Y=f(a)+\varepsilon$$

where *a* ∈ R^*D*^ is an input vector according to a bounded distribution $\Omega$ whereas *Y* ∈ R is an output vector, and variance $\rho ={\sigma }^{2}\in R$. MPMR sets an approximation function $\hat{f}$, where for ${x}_{i}$ generated from $\Omega$:

12 $$\hat{Y}=\hat{f}(a)$$

The bounds are determined by model based on minimum probability ($\omega$), that $\hat{f}(a)$ is within $\varepsilon$ of *Y*^79^:

13 $$\omega =\mathit{inf}\hspace{0.33em}\mathit{Pr}\left\{\left|\hat{Y}\hspace{0.33em}-Y\right|\le \varepsilon \right\}$$

By minimax probability presented in Eq. (13), the prediction power of a true regression is calculated by a bound-on minimax probability. Hence, deducing $\omega$ within $\varepsilon$ of the true function^80^. The MPMR model is built based on kernel function,

14 $$\hat{Y}=\hat{f}(a)={\sum }_{i=1}^{N}{\chi }_{i}K({a}_{i},{a}_{j})+\varphi$$

where ${K}_{i,j}=\Delta \left({a}_{i},{a}_{j}\right)$ is the kernel function based on Mercer condition, ${a}_{i}$ is from the learning data, ${\chi }_{i}$ and $\varphi$ are the output parameters. The schematic view of MPMR model is shown in Fig. 2d.

#### Multi-scale standardized precipitation index (SPI)

The SPI quantifies the wet and dry scenarios based on statistical probability theory. Before developing the forecasting models, the multi-scale SPI index was calculated from rainfall (*RnF*) time-series^39^ using a gamma distribution function ($g(RnF)$):

15 $$g\left(RnF\right)=\frac{1}{{\beta }^{\alpha }\Gamma \left(\alpha \right)}{\left(RnF\right)}^{\alpha -1}{e}^{-\frac{RnF}{\beta }}\mathrm{for }RnF>0$$

where *α* and *β* are the parameters determined by maximum likelihood estimator, and $\Gamma (\alpha )$ is the mathematical gamma function. The cumulative probability ($G(RnF)$) is defined as:

16 $$G(RnF)={\int }_{0}^{\infty }g(RnF)dRnF=\frac{1}{{\beta }^{\alpha }\Gamma (\alpha )}{\int }_{0}^{Rnf}{x}^{\alpha -1}{e}^{-RnF/{\beta }_{dRnF}}$$

By substituting *t* = *RnF*/*β*, Eq. (16), $G(RnF)$ becomes:

17 $$G(RnF)=\frac{1}{\Gamma (\alpha )}{\int }_{0}^{RnF}{t}^{\alpha -1}{e}^{-t}dt$$

**Figure 3:**
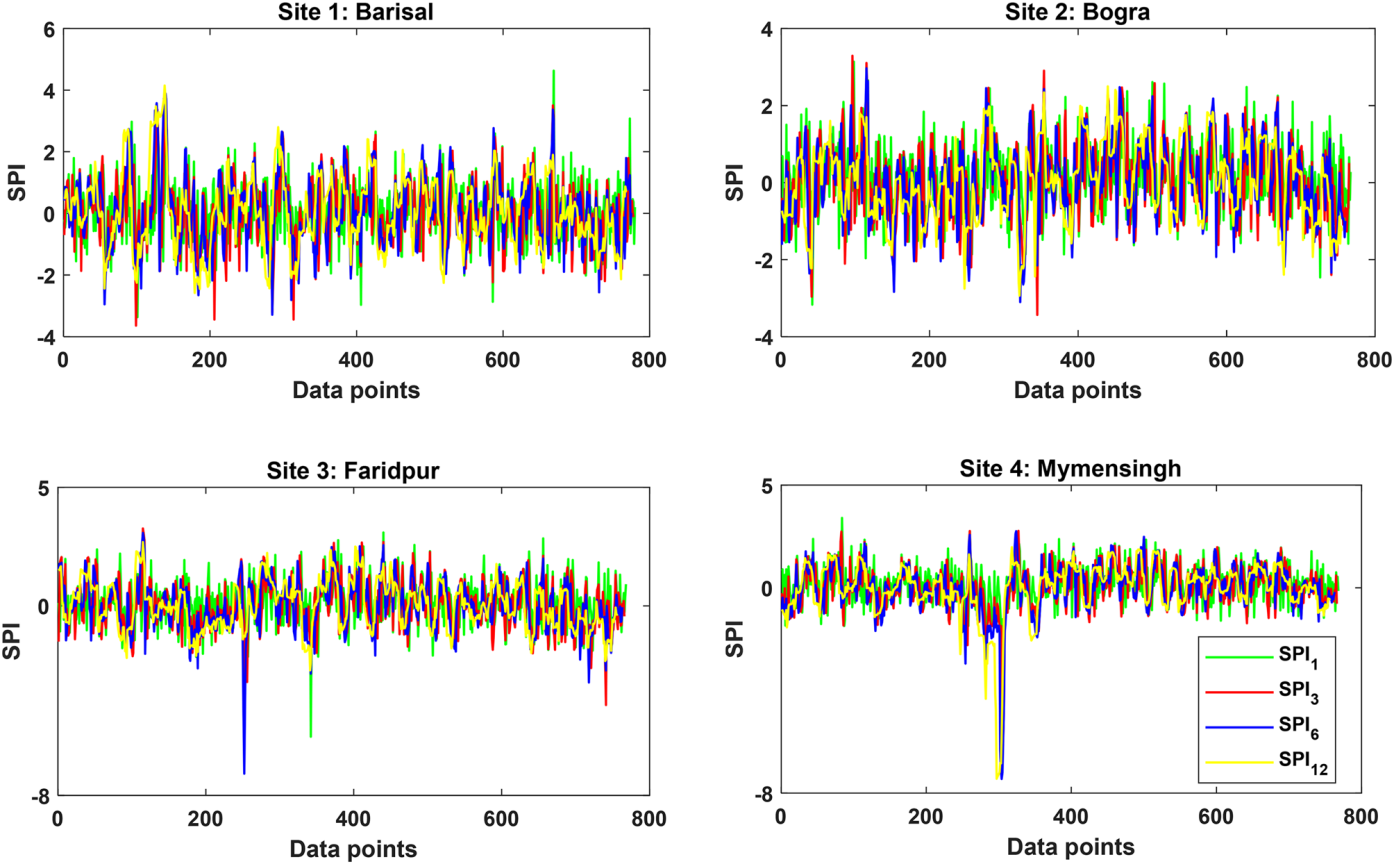
The time series of the generated SPI for the four meteorological stations.

The cumulative probability reduces to the following form when *RnF* = 0:

18 $$H\left( {RnF} \right) \, = p + \, \left( {{1} - p} \right)G\left( {RnF} \right)$$

with *p* represents the probability of zero which determines the SPI index as:

19 $$SPI=\left\{\begin{array}{c}+\left(n-\frac{{\varepsilon }_{0}+{\varepsilon }_{1}n+{\varepsilon }_{2}{n}^{2}}{1+{\omega }_{1}n+{\omega }_{2}{n}^{2}+{\omega }_{3}{n}^{3}}\right),\hspace{1em}0.5<H(RnF)\le 1.0\\ -\left(n-\frac{{\varepsilon }_{0}+{\varepsilon }_{1}n+{\varepsilon }_{2}{n}^{2}}{1+{\omega }_{1}n+{\omega }_{2}{n}^{2}+{\omega }_{3}{n}^{3}}\right),\hspace{1em}0<H(RnF)\le 0.5\end{array}\right.$$

where ${\varepsilon }_{0},{\varepsilon }_{1},{\varepsilon }_{2},{\varepsilon }_{3}$, ${\omega }_{1},{\omega }_{2}$ and ${\omega }_{3}$ are arbitrary constants with magnitudes: ${\varepsilon }_{0}=2.515517$, ${\varepsilon }_{1}=0.802853$, ${\varepsilon }_{3}=0.010328$, ${\omega }_{1}=1.432788$, ${\omega }_{2}=0.189269$ and ${\omega }_{3}=0.001308$^39^. Drought is categorized into three as moderate = (− 1*.*5 < SPI ≤ 1*.*0), severe = (− 2.0 < *SPI* ≤  − 1.5), and extreme = (SPI ≤  − 2*.*0). The time series of the generated SPI for different scales at all the four meteorological stations are presented in Fig. 3.

### Models development and evaluation metrics

**Figure 4:**
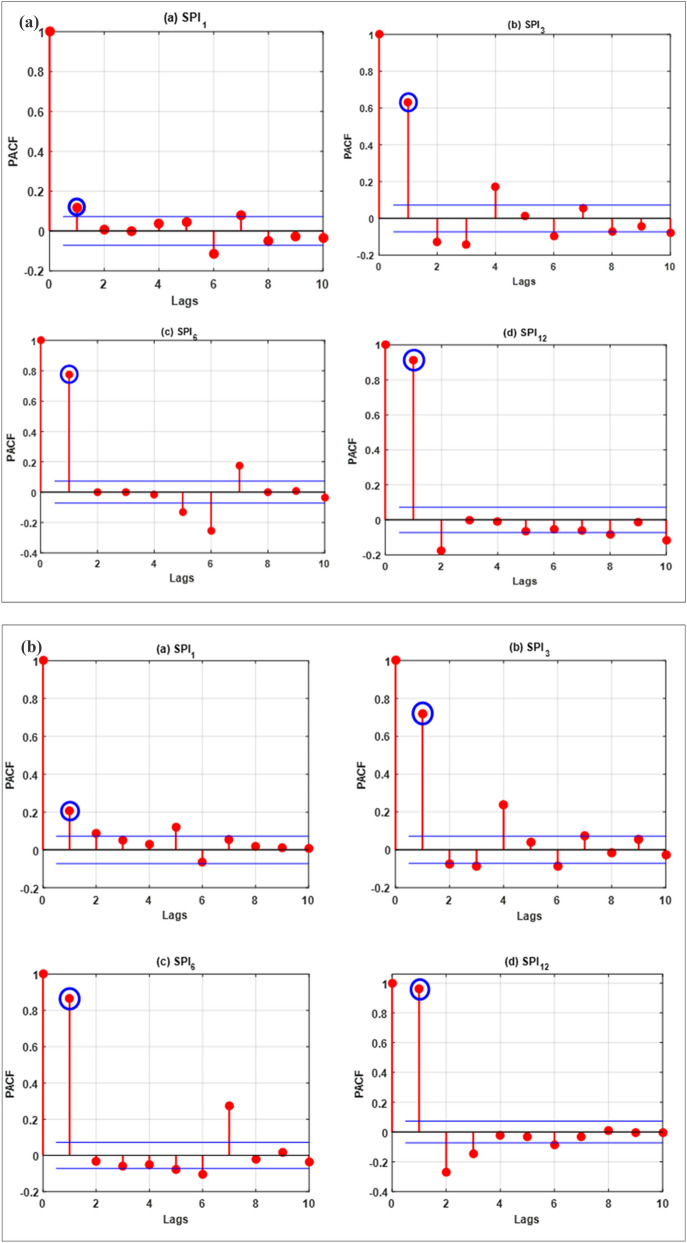

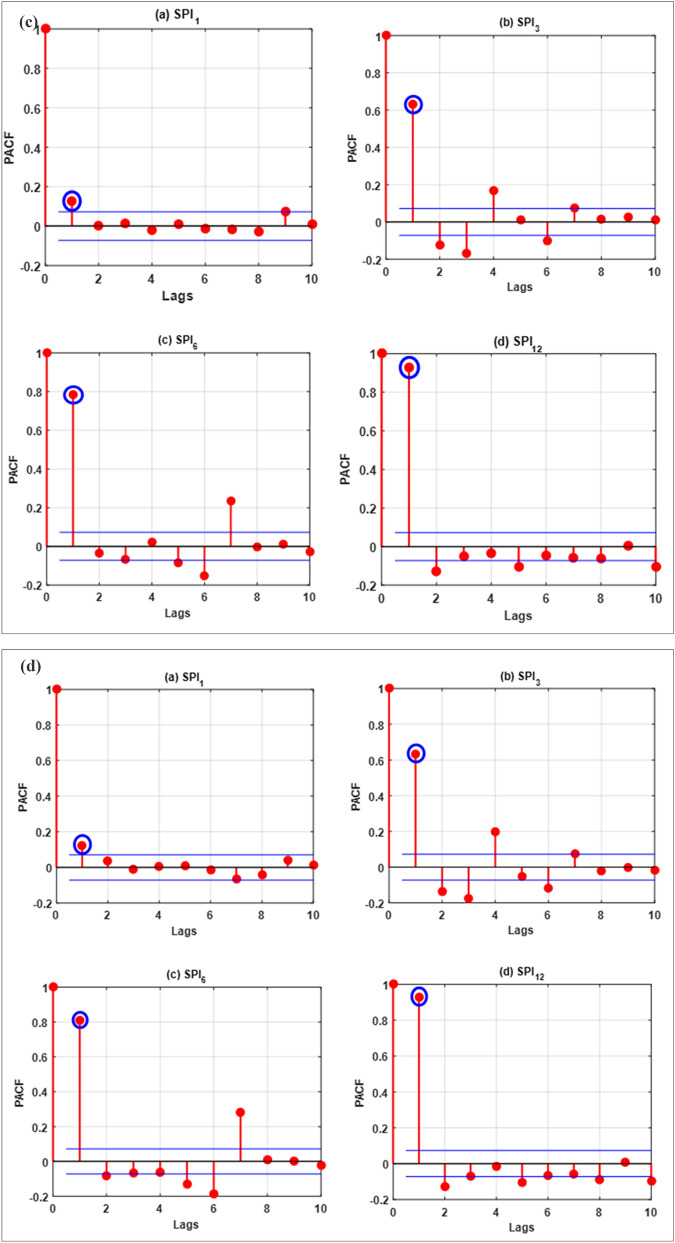
The correlation statistics of the lags for all the inspected meteorological time series data (**a**) Barisal station, (**b**) Bogra station, (**c**) Faridpur station and (**d**) Mymensingh station.
Step 2: Normalization process
  The normalization process is essential for data scaling to solve the problem of high variation in data. In this study, data were scaled between [0, 1] using Eq. (17):
  22 $$SP{I}_{norm}=\frac{\left(SPI-SP{I}_{min}\right)}{\left(SP{I}_{max}-SP{I}_{min}\right)}$$
  In Eq. (17), $SPI$ represents the input/output, $SP{I}_{min}$ is the minimum SPI value, $SP{I}_{max}$ is the maximum SPI value and $SP{I}_{norm}$ is the value corresponding normalized numeric.
Step 3: Applying data intelligent methods.
  The data were divided into 70–30% for the training and testing the models. The number of trees (1000) was defined before developing the RF model. Different activation functions (hardlim, radial basis, sine, sigmoid) were evaluated to determine the best activation function for various numbers of unseen neurons in the range from 1 to  50 before development of OSELM and ELM models. The size of the block was set to 100 for OSELM. The significant lags at (t-1) were used in M5tree and MPMR models to forecast SPI_1_, SPI_3_, SPI_6_ and SPI_12_. For the development of MPMR model, the linear, polynomial and Gaussian kernels were used.

The forecasting models were developed in MATLAB R2016b programming environment (The Math Works Inc. USA). By operating Pentium 4, 2.93 GHz dual-core Central Processing Unit, all the simulations were obtained. Historical rainfall data was used to compute the SPI for 64 years. The training phase was built using 75% of the data (1949–1997) while the testing was conducted with the remaining 25% data (1998–2013). These forecasting ML models were developed using the steps as follow:

Step 1: Computing Partial autocorrelation functions (PACFs) of SPI_1_, SPI_3_, SPI_6_ and SPI_12_ to estimate the significant lags. The PACF was estimated for SPI_1_, SPI_3_, SPI_6_ and SPI_12_ using following equation^81^:
  20 $${\sigma }_{l}=\frac{\frac{1}{n-l}{\sum }_{t=l+1}^{n}\left(SP{I}_{t}-\overline{SPI}\right)\left(SP{I}_{t-l}-\overline{SPI}\right)}{\sqrt{\frac{1}{n}{\sum }_{t=1}^{n}\left(SP{I}_{t}-\overline{SPI}\right)}\sqrt{\frac{1}{n-l}{\sum }_{t=l+1}^{n}\left(SP{I}_{t-l}-\overline{SPI}\right)}}$$
  where $SP{I}_{t}$ is the observed series; $l = 0,1,2, \ldots$ for $t = 1,2,3, \ldots ,n$; and $\overline{SPI}$ denotes mean SPI. The PACF is defined as:
  21 $$SP{I}_{t}={\Gamma }_{21}SP{I}_{t-1}+{\Gamma }_{22}SP{I}_{t-1}+{E}_{t}$$
  where ${\Gamma }_{22}$ indicates that the order 2 PACF is expected to yield. A greater positive value of PACF indicates a good input for drought forecasting model development. Figure 4a–d present the statistical correlations estimated for the investigated stations.

Several performance metrics were computed for the evaluation of model performance including mean square error (*MSE*), correlation coefficient (*R*), Willmott’s Index of agreement (*WI*), Nash Sutcliffe efficiency (*NSE*), root mean square (*RMSE*), mean absolute error (*MAE*) and Legates and McCabe Index (*LM*)^82–85^. The mathematical expression of the metrics are as follows:

23 $$MSE=\sum_{i=1}^{n}\frac{{({SPI}_{o}-{SPI}_{f})}^{2}}{n}$$

24 $$R=\frac{{\sum }_{i=1}^{n}({SPI}_{o}-{\overline {SPI}}_{o})({SPI}_{f}-{\overline {SPI}}_{f})}{\sqrt{{\sum }_{i=1}^{n}{({SPI}_{o}-{\overline {SPI}}_{o})}^{2}}\sqrt{{\sum }_{i=1}^{n}{({SPI}_{f}-{\overline {SPI}}_{f})}^{2}}}$$

25 $$WI=1-\frac{{\sum }_{i=1}^{n}{({SPI}_{o}-{SPI}_{f})}^{2}}{{\sum }_{i=1}^{n}{\left(\left|{SPI}_{f}-{\overline {SPI}}_{o}\right|+\left|{SPI}_{o}-{\overline {SPI}}_{o}\right|\right)}^{2}}$$

26 $$NSE=1-\frac{{\sum }_{i=1}^{n}{\left({SPI}_{o}-{SPI}_{f}\right)}^{2}}{{\sum }_{i=1}^{n}{\left({SPI}_{o}-{\overline {SPI}}_{o}\right)}^{2}}$$

27 $$RMSE=\sqrt{\sum_{i=1}^{n}\frac{{({SPI}_{o}-{SPI}_{f})}^{2}}{n}}$$

28 $$MAE=\frac{{\sum }_{i=1}^{n}\left|{SPI}_{o}-{SPI}_{f}\right|}{n}$$

29 $$LM=1-\frac{{\sum }_{i=1}^{n}\left|{SPI}_{o}-{\overline {SPI}}_{f}\right|}{{\sum }_{i=1}^{n}\left|{SPI}_{o}-{\overline {SPI}}_{o}\right|}$$

where ${SPI}_{o}$ and ${SPI}_{f}$ are observed and predicted SPI values. ${\overline {SPI}}_{o}$ and ${\overline {SPI}}_{f}$ are the mean of the observed and predicted SPI values. *n* is the number of sample in the dataset. *RMSE* provides more weight to higher different between observed and modelled SPIs in estimating model error and therefore, it provides a better estimation of model performance and most widely used by modellers to derive the conclusion.

## Results and discussion

Drought prediction for Bangladesh was conducted in this study using the SPIs for different time-scales (1, 3, 6, 9 and 12 months) at four meteorological stations distributed over the country (e.g. Barisal, Bogra, Faridpur and Mymensingh). The prediction process was conducted using relatively new ML models such as RF, MPMR, M5tree, ELM and OSELM. To build the predictive models, the correlated antecedent SPI values were used as inputs.

The employed predictive models were trained using 50-year monthly data (1949–1997) while the testing was conducted using 16-year data (1998–2013) at all the four stations. To enhance the accuracy in prediction of SPI, all predictive variables were standardized in a range of 0 to 1. Adequacy of each predictive model was quantified using performance indices such as *MSE, R, WI, NSE*, *RMSE*, *MAE* and *LM* (Eqs. (18–24)). The predictive models were compared based on their performance during the testing phase.

The performance of the models in terms of statistical metrics is shown in Tables 1, 2, 3, 4. The results revealed that RF is the best predictive model for SPI-1 ($RMSE=0.43{-}0.54$) at all stations while for the other scales of SPI such as SPI-3 ($RMSE=0.2{-}0.72$), SPI-6 ($RMSE=0.09{-}0.22$) and SPI-12 ($RMSE=0.03{-}0.08$) both ELM and OSELM showed superiority compared to others. However, the obtained results using ELM ($RMSE=0.37$) showed a bit higher accuracy compared to OSELM ($RMSE=0.72$) at Faridpur station for SPI-3. The M5tree ($RMSE=0.4{-}0.94$) and MPMR ($RMSE=0.37{-}0.84$) showed the lowest performance in predicting SPI for all the scales. Hence, it can be concluded that ELM is the best performing model while M5tree has the lowest accuracy in prediction of SPI. All predictive models showed better accuracy in predicting SPI for higher scales. This evidenced the potential of non-tuned extreme learning machine model in forecasting droughts in Bangladesh. Recently, the feasibility of the ELM model is successfully implemented for drought indices simulation in many other studies^44,86,87^.

**Table 1:** The statistical performance of the prediction models for different SPI scales at Barisal station.

Predictive models	*MSE*	*R*	*WI*	*NSE*	*RMSE*	*MAE*	*LM*
**SPI_1**							
RF	**0.327**	**0.845**	**0.732**	**0.661**	**0.57**	**0.46**	**0.414**
MPMR	0.986	0.031	− 0.132	− 0.021	0.99	0.79	− 0.018
M5tree	1.057	0.011	− 0.081	− 0.095	1.03	0.81	− 0.042
ELM	0.633	0.625	0.128	0.344	0.80	0.66	0.150
OSELM	0.720	0.963	0.165	0.254	0.85	0.66	0.152
**SPI_3**							
RF	0.224	0.894	0.832	0.769	0.47	0.37	0.506
MPMR	0.673	0.566	0.419	0.306	0.82	0.61	0.174
M5tree	0.882	0.448	0.317	0.090	0.94	0.68	0.082
ELM	**0.111**	**0.990**	**0.906**	**0.886**	**0.33**	**0.26**	**0.655**
OSELM	0.121	0.985	0.893	0.875	0.35	0.27	0.634
**SPI_6**							
RF	0.145	0.923	0.891	0.842	0.38	0.30	0.603
MPMR	0.489	0.699	0.629	0.468	0.70	0.52	0.300
M5tree	0.575	0.663	0.613	0.374	0.76	0.57	0.230
ELM	**0.024**	**0.997**	**0.983**	**0.974**	**0.15**	**0.11**	**0.856**
OSELM	0.024	0.998	0.983	0.974	0.15	0.12	0.845
**SPI_12**							
RF	0.058	0.967	0.960	0.934	0.24	0.17	0.773
MPMR	0.205	0.876	0.857	0.763	0.45	0.29	0.613
M5tree	0.209	0.872	0.853	0.760	0.46	0.31	0.594
ELM	**0.004**	**0.99998**	**0.997**	**0.996**	**0.06**	**0.05**	**0.935**
OSELM	0.004	0.99988	0.997	0.995	0.07	0.06	0.924

Boldface results are the best prediction accuracy.

**Table 2:** The statistical performance of the prediction models for different SPI scales at Bogra station.

Predictive models	*MSE*	*R*	*WI*	*NSE*	*RMSE*	*MAE*	*LM*
**SPI_1**							
RF	**0.294**	**0.815**	**0.671**	**0.590**	**0.54**	**0.45**	**0.35**
MPMR	0.714	0.098	− 0.051	0.006	0.84	0.68	0.007
M5tree	0.856	− 0.006	− 0.124	− 0.192	0.93	0.73	− 0.065
ELM	0.572	0.800	0.182	0.204	0.76	0.60	0.128
OSELM	0.571	0.815	0.202	0.205	0.76	0.61	0.118
**SPI_3**							
RF	0.169	0.907	0.877	0.809	0.41	0.33	0.560
MPMR	0.551	0.616	0.516	0.377	0.74	0.60	0.217
M5tree	0.619	0.565	0.497	0.300	0.79	0.62	0.183
ELM	**0.121**	**0.996**	**0.897**	**0.864**	**0.35**	**0.28**	**0.630**
OSELM	0.113	0.994	0.905	0.872	0.34	0.29	0.616
**SPI_6**							
RF	0.132	0.930	0.915	0.859	0.36	0.28	0.646
MPMR	0.410	0.750	0.716	0.561	0.64	0.48	0.384
M5tree	0.456	0.725	0.698	0.512	0.68	0.51	0.346
ELM	**0.043**	**0.998**	**0.971**	**0.954**	**0.21**	**0.17**	**0.784**
OSELM	0.049	0.995	0.966	0.947	0.22	0.19	0.753
**SPI_12**							
RF	0.048	0.975	0.975	0.950	0.22	0.16	0.807
MPMR	0.156	0.914	0.913	0.836	0.39	0.26	0.678
M5tree	0.170	0.908	0.906	0.821	0.41	0.27	0.663
ELM	**0.006**	**1.000**	**0.997**	**0.993**	**0.08**	**0.06**	**0.924**
OSELM	0.006	1.000	0.997	0.994	0.08	0.06	0.923

Boldface results are the best prediction accuracy.

**Table 3:** The statistical performance of the prediction models for different SPI scales at Faridpur station.

Predictive models	*MSE*	*R*	*WI*	*NSE*	*RMSE*	*MAE*	*LM*
**SPI_1**							
RF	**0.355**	**0.809**	**0.678**	**0.577**	**0.596**	**0.469**	**0.347**
MPMR	0.851	0.154	− 0.186	0.005	0.923	0.730	− 0.008
M5tree	0.981	0.078	− 0.158	− 0.146	0.990	0.793	− 0.095
ELM	0.692	0.977	0.005	0.191	0.832	0.658	0.091
OSELM	0.639	0.742	0.244	0.253	0.800	0.664	0.084
**SPI_3**							
RF	0.241	0.878	0.838	0.750	0.491	0.395	0.487
MPMR	0.647	0.584	0.454	0.328	0.804	0.629	0.184
M5tree	0.740	0.516	0.440	0.230	0.860	0.672	0.128
ELM	**0.135**	**1.000**	**0.893**	**0.859**	**0.368**	**0.291**	**0.622**
OSELM	0.511	0.696	0.510	0.469	0.715	0.324	0.579
**SPI_6**							
RF	0.116	0.926	0.913	0.850	0.341	0.261	0.617
MPMR	0.362	0.736	0.691	0.534	0.602	0.453	0.336
M5tree	0.358	0.739	0.695	0.540	0.598	0.453	0.335
ELM	**0.041**	**0.998**	**0.966**	**0.948**	**0.201**	**0.136**	**0.800**
OSELM	0.051	0.994	0.955	0.934	0.226	0.144	0.788
**SPI_12**							
RF	0.049	0.963	0.957	0.925	0.222	0.155	0.765
MPMR	0.140	0.888	0.878	0.786	0.374	0.252	0.619
M5tree	0.162	0.872	0.858	0.752	0.403	0.281	0.575
ELM	**0.003**	**0.999**	**0.997**	**0.995**	**0.058**	**0.040**	**0.940**
OSELM	0.004	0.999	0.997	0.994	0.062	0.044	0.934

Boldface results are the best prediction accuracy.

**Table 4:** The statistical performance of the prediction models for different SPI scales at Mymensingh station.

Predictive models	*MSE*	*R*	*WI*	*NSE*	*RMSE*	*MAE*	*LM*
**SPI_1**							
RF	**0.184**	**0.854**	**0.796**	**0.692**	**0.429**	**0.340**	**0.438**
MPMR	0.634	− 0.003	0.068	− 0.071	0.796	0.624	− 0.039
M5tree	0.631	0.063	0.031	− 0.066	0.795	0.626	− 0.043
ELM	0.292	1.000	0.570	0.507	0.541	0.422	0.297
OSELM	0.366	0.890	0.449	0.383	0.605	0.477	0.205
**SPI_3**							
RF	0.131	0.890	0.851	0.771	0.362	0.294	0.525
MPMR	0.408	0.560	0.522	0.289	0.638	0.525	0.152
M5tree	0.481	0.508	0.501	0.161	0.693	0.579	0.064
ELM	**0.041**	**0.998**	**0.953**	**0.928**	**0.203**	**0.155**	**0.749**
OSELM	0.052	0.993	0.941	0.910	0.228	0.177	0.713
**SPI_6**							
RF	0.075	0.926	0.902	0.847	0.274	0.211	0.631
MPMR	0.267	0.699	0.686	0.454	0.517	0.387	0.322
M5tree	0.255	0.704	0.674	0.478	0.505	0.379	0.335
ELM	**0.008**	**0.999**	**0.991**	**0.983**	**0.090**	**0.065**	**0.886**
OSELM	0.011	1.000	0.986	0.977	0.107	0.090	0.842
**SPI_12**							
RF	0.020	0.975	0.975	0.950	0.140	0.100	0.809
MPMR	0.063	0.917	0.922	0.840	0.251	0.163	0.688
M5tree	0.063	0.917	0.923	0.839	0.251	0.163	0.688
ELM	**0.001**	**1.000**	**0.999**	**0.997**	**0.033**	**0.029**	**0.945**
OSELM	0.002	0.999	0.998	0.996	0.042	0.034	0.934

Boldface results are the best prediction accuracy.

**Figure 5:**
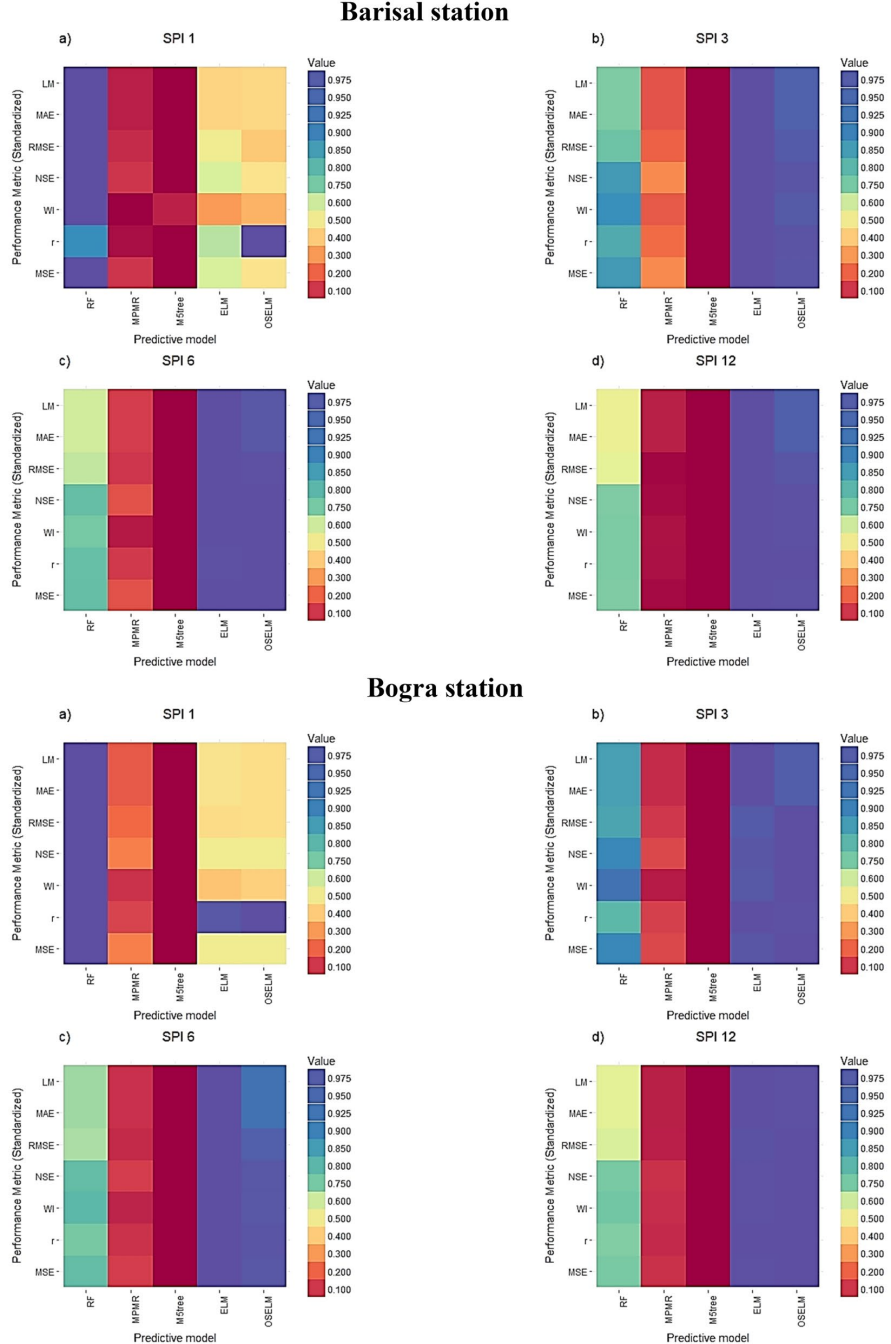

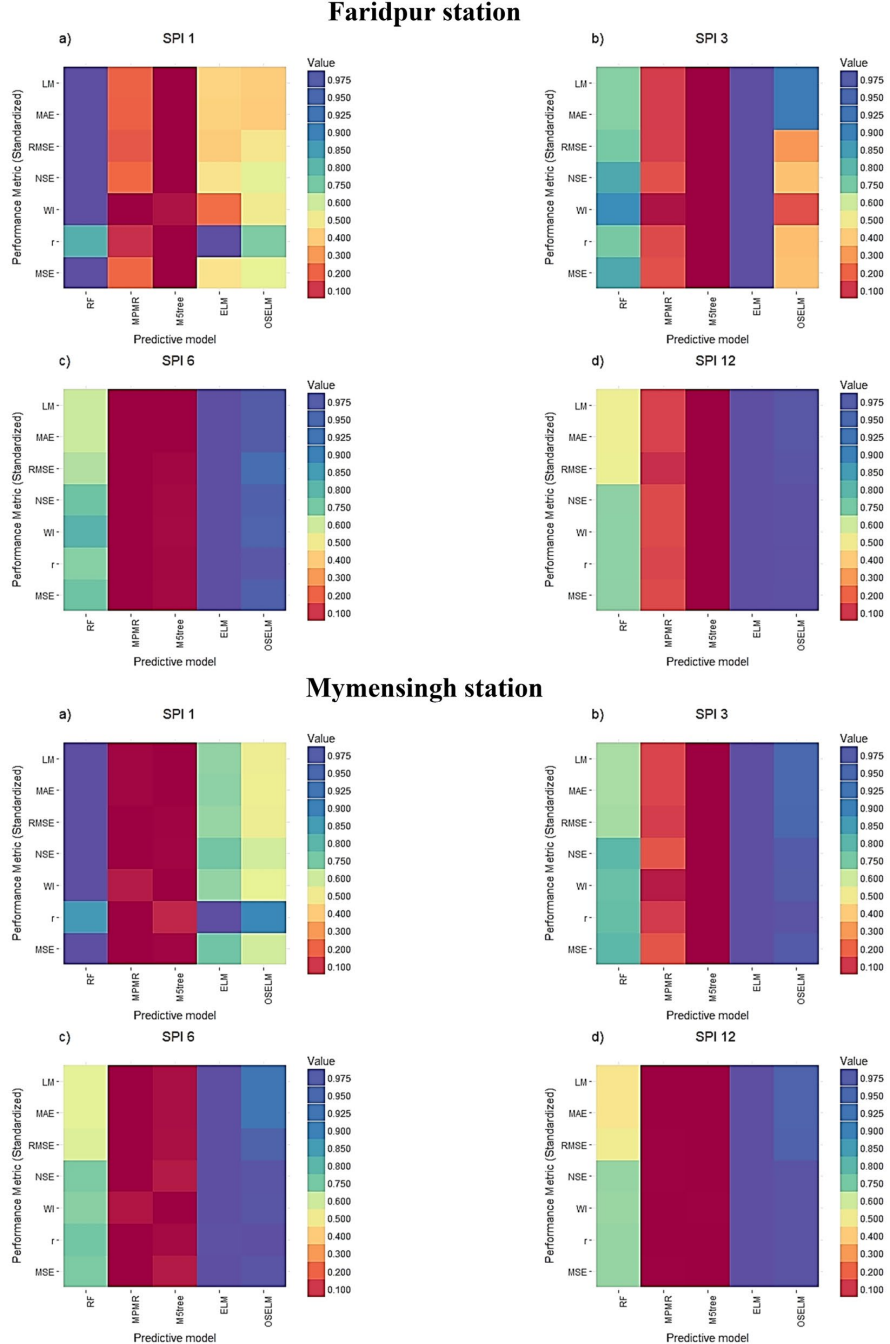
Heat map showing the performance of different predictive models in terms of different statistics metrics at the four investigated meteorological stations and multiple SPI scales.

The graphical evaluation and assessment among the predictive models in term of standardized performance indices are depicted in a form of Heatmap diagram in Fig. 5. The dark blue color in the figure represents the best statistical performance while dark red color represents the worst performance. It can be seen from the figure that ELM and OSELM showed the best performance in term of all metrics for all SPIs except SPI-1. Furthermore, ELM showed the highest performance compared to other models at all stations. Besides, the maximum number of dark red cells (worst predictive model) was shown by M5tree model.

**Figure 6:**
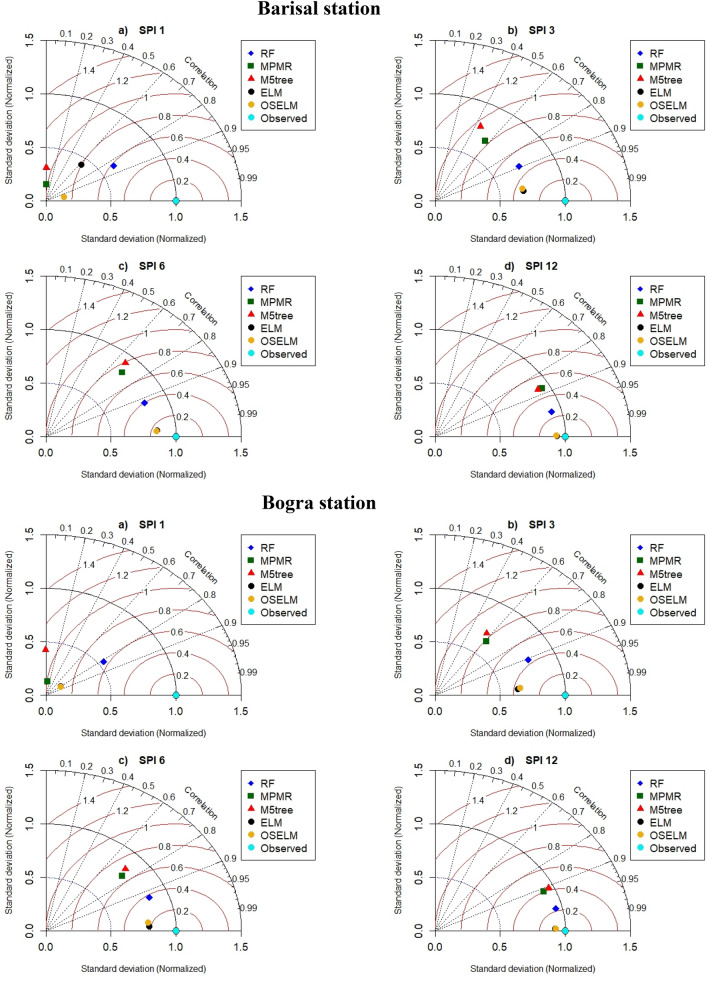

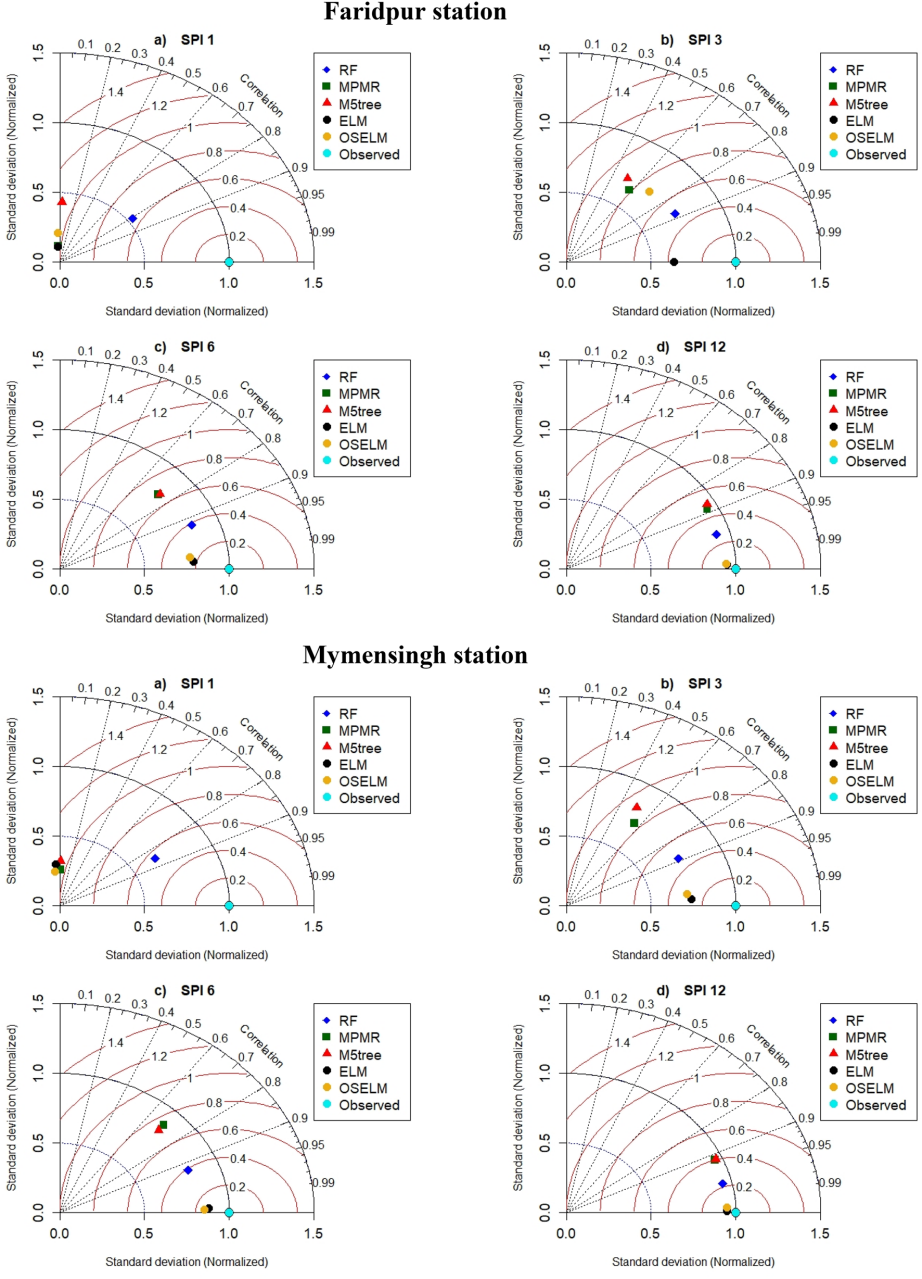
Taylor diagram presentation of the performance of the predictive models at four investigated meteorological stations and multiple SPI scales.

Taylor diagram is another graphical presentation which was used to make a comparison among the employed predictive models (Fig. 6). The results of Taylor diagrams indicated good consistency with the obtained performance indices. Figure 6 shows that the highest agreement exists between the RF prediction (blue rectangular) and observed SPI-1 at all the stations. For other SPI scales, ELM and OSELM showed relatively same results which indicate their superiority compared to other predictive models. These models provided the lowest normalized *RMSE* (less than 0.4), the highest correlations (more than 0.95) and the lowest variation (within 0.6–0.9). However, ELM provided better results for SPI-3 in Faridpur station compared to OSELM while at other stations and SPI scales the results were found the same.

**Figure 7:**
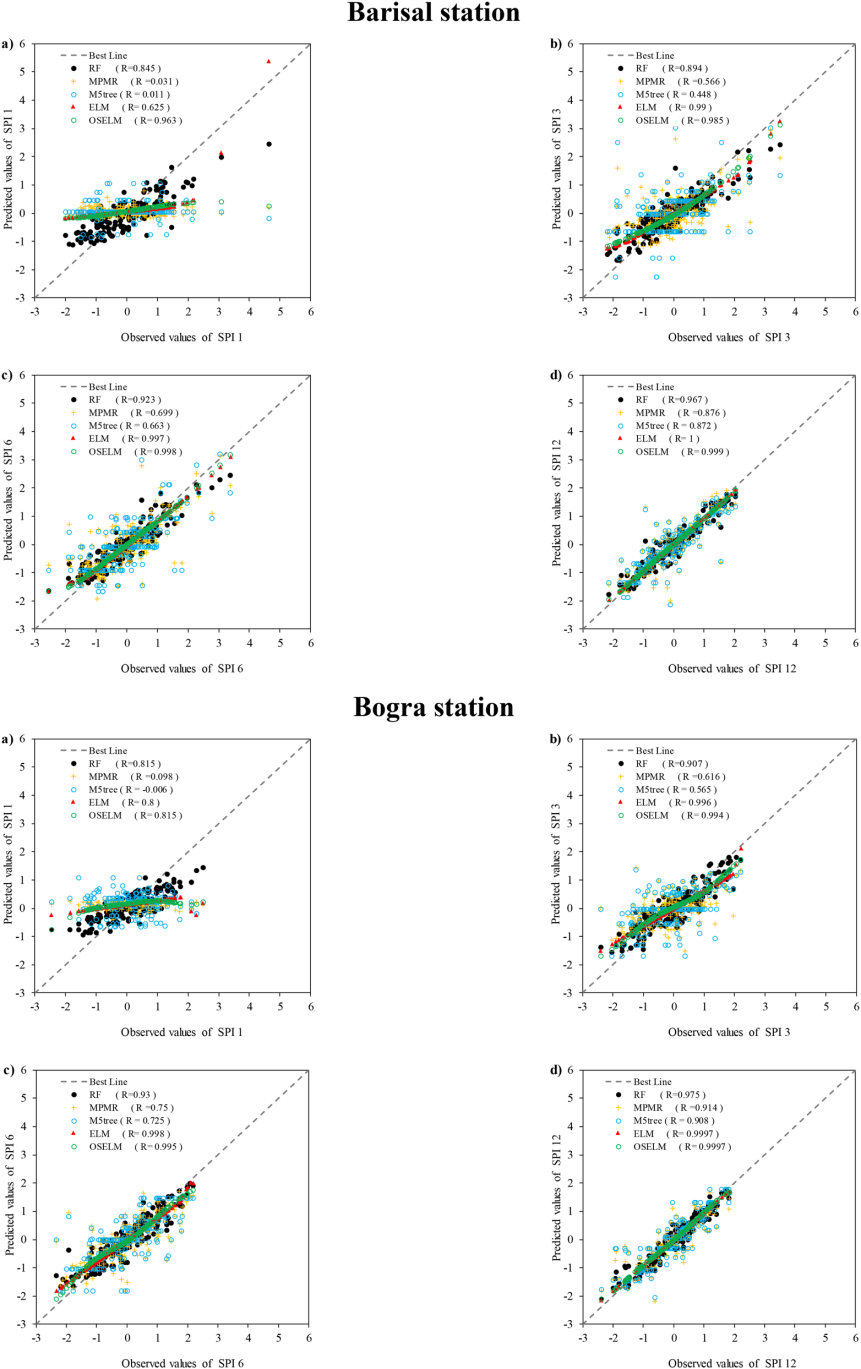

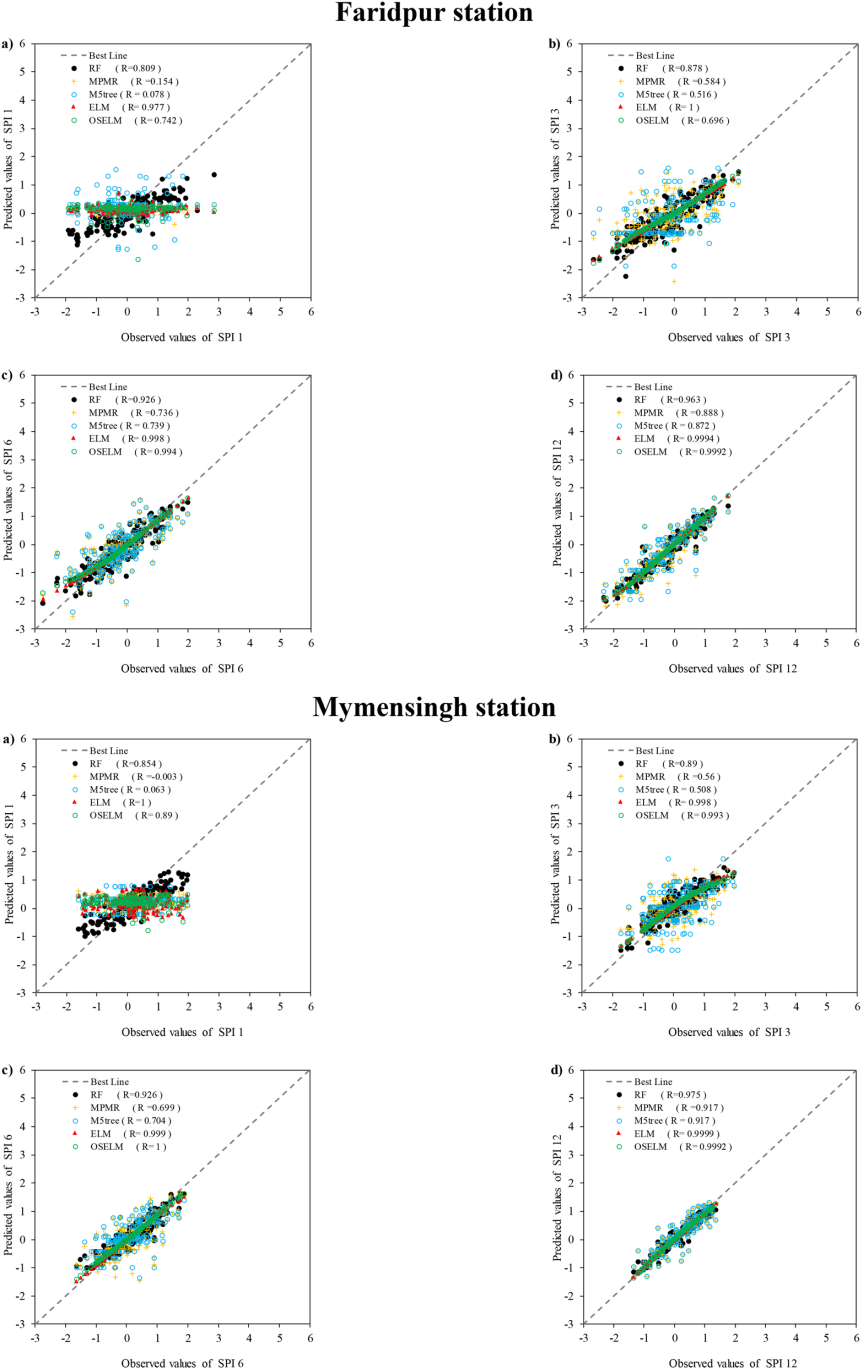
The scatter plot between the observed and predicted SPI obtained using the predictive models at the four investigated meteorological stations and multiple SPI scales.

Scatter plots shows the linear correlation between observed and predicted SPI values at all stations (Fig. 7). The results revealed that the prediction of all the predictive models except M5tree and MPMR have a high correlation with observation. Most of the predicted points are aligned to the perfect line (45° line) which shows a significant performance of prediction models. Based on the obtained values of correlation coefficients, it can be seen that OSELM ($R=0.81{-}0.96$) has better correlation for SPI-1 at all stations except Faridpur ($R=0.74$). However, it can be concluded that the best correlation for SPI-1 was attained using OSELM and the worst results by M5tree ($R=-0.006{-}0.011$). For other SPI scales, it is clear that ELM provided a higher accuracy compared to other models. However, there was no significant difference between the results obtained using ELM ($R=0.98{-}0.999$) and OSELM ($R=0.69{-}0.999$) at all the considered stations. Therefore, both the ELM and OSELM models indicated a higher correlation between the observed and predicted SPI for different scales in comparison with RF, MPMR and M5tree models. The M5tree ($R=-0.006{-}0.011$) provided the lowest correlation coefficient. Overall, it can be remarked that both ELM and OSELM models have adequate capability in SPI prediction.

**Figure 8:**
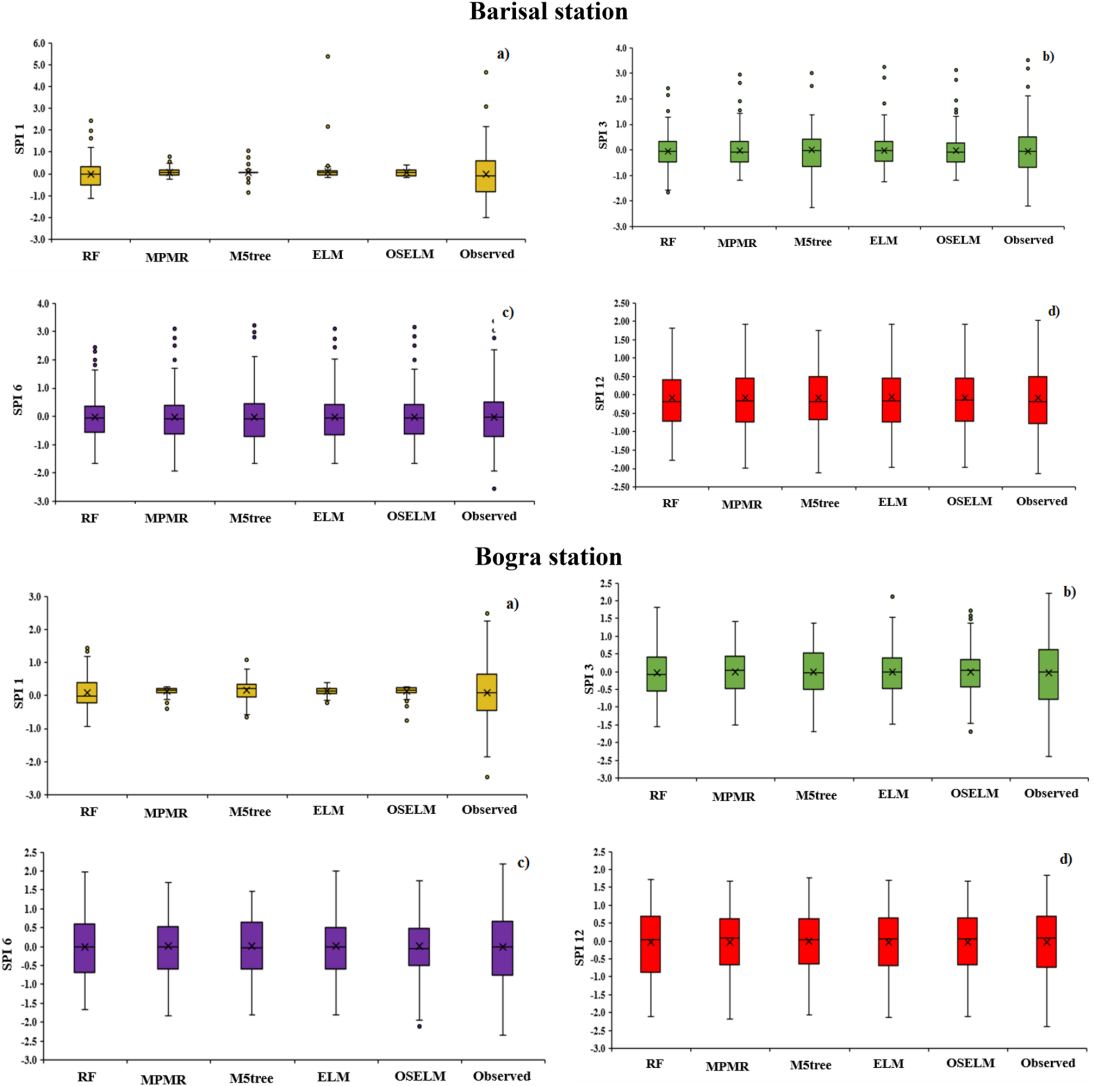

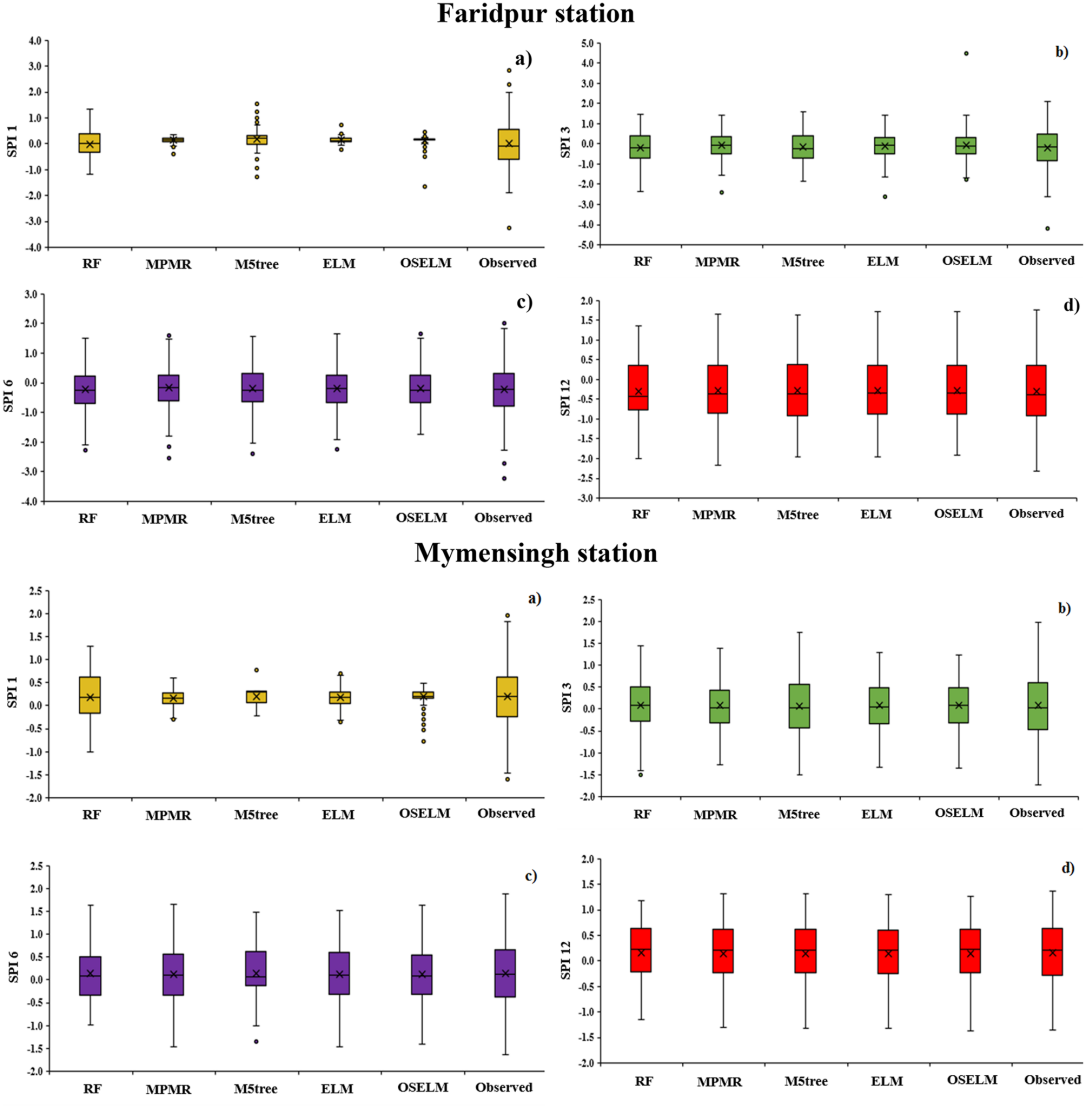
Box plot presentation of the performance of the predictive models at the four investigated meteorological stations and multiple SPI scales.

To assess the uncertainty in SPI prediction, 25%, 50% and 75% quantile values of the observed and predicted SPI are presented using boxplots in Fig. 8. The figure shows that the variability in SPI-1 values could not be simulated by any of the models adequately. Many predicted SPI-1 values were found fluctuating near to zero (narrow range) while the observed values have a wide range [− 2 to 2]. However, the RF model showed better accuracy to simulate variability and quartiles of SPI-1 compared to others. All predictive models were found to show better results in simulating SPI quantiles of other SPI scales, especially for their higher orders. Overall, the ELM and OSELM provided the highest accuracy to simulate the variability of SPI values while M5tree showed the worst. Hence, it can be remarked that M5tree is not suitable for prediction of SPI in any regions of Bangladesh.

## Conclusions

The current research is attempted to investigate the feasibility of newly developed ML models to forecast multiple scales of SPI drought index over Bangladesh. The developed predictive models are inspected on the monthly scale of rainfall data for the period of 1949–2013 at four different meteorological stations. The predictors of the forecasting models were accomplished using the potential of the statistical auto-correlation method. The attained forecasting results demonstrate consistency in results obtained using ELM for the 3-, 6- and 12-month SPI. It showed the minimal *RMSE* (0.33, 0.15 and 0.06), (0.35, 0.21 and 0.08), (0.36, 0.20 and 0.05) and (0.020, 0.09 and 0.02) at Barisal, Bogra, Faridpur and Mymensingh meteorological stations in predicting the SPI-3, SPI-6 and SPI-12, respectively. Whereas, the RF showed the best performance for one-month SPI with minimal *RMSE* values of 0.57, 0.45, 0.59 and 0.42 for those four stations. The results indicate the potential of the models to be employed for drought forecasting in Bangladesh for the mitigation of drought impacts. In future, other ML models can be employed to evaluate their performance in forecasting droughts in Bangladesh. Besides, different optimization methods can be used for the optimization of ML model parameters to improve their prediction capability.

## Data Availability

The used dataset in this research are available upon request from the corresponding author.
